# Receptor-Mediated Endocytosis of α-Galactosidase A in Human Podocytes in Fabry Disease

**DOI:** 10.1371/journal.pone.0025065

**Published:** 2011-09-19

**Authors:** Thaneas Prabakaran, Rikke Nielsen, Jakob V. Larsen, Søren S. Sørensen, Ulla Feldt- Rasmussen, Moin A. Saleem, Claus M. Petersen, Pierre J. Verroust, Erik I. Christensen

**Affiliations:** 1 Section of Cell Biology, Department of Anatomy, Aarhus University, Aarhus, Denmark; 2 Department of Medical Biochemistry, Aarhus University, Aarhus, Denmark; 3 Department P, Rigshospitalet, Copenhagen, Denmark; 4 Department of Medical Endocrinology, Rigshospitalet, Copenhagen, Denmark; 5 Children's Renal Unit and Academic Renal Unit, University of Bristol, Southmead Hospital, Bristol, United Kingdom; 6 UMRS 592, Institut de la Vision, Paris, France; INSERM, France

## Abstract

Injury to the glomerular podocyte is a key mechanism in human glomerular disease and podocyte repair is an important therapeutic target. In Fabry disease, podocyte injury is caused by the intracellular accumulation of globotriaosylceramide. This study identifies in the human podocyte three endocytic receptors, mannose 6-phosphate/insulin-like growth II receptor, megalin, and sortilin and demonstrates their drug delivery capabilities for enzyme replacement therapy. Sortilin, a novel α-galactosidase A binding protein, reveals a predominant intracellular expression but also surface expression in the podocyte. The present study provides the rationale for the renal effect of treatment with α-galactosidase A and identifies potential pathways for future non-carbohydrate based drug delivery to the kidney podocyte and other potential affected organs.

## Introduction

Fabry disease is an X-linked lysosomal disorder that results from mutations of the gene (*GLA*) that encodes α-galactosidase A (α-Gal A) [1], [2], [3]. The enzymatic defect leads to progressive lysosomal accumulation of globotriaosylceramide (GL-3) and related glycosphingolipids in the kidney and other tissues [3]. Glycosphingolipid accumulates over time leading to kidney failure, cerebrovascular manifestations, and heart failure, and eventually premature death [4].

Nephropathy is a dominant feature in Fabry disease and impairment of renal function occurs due to progressive accumulation of GL-3 in a variety of renal cells [3], [5], [6], [7], [8]. End-stage renal failure usually occurs in the third to fifth decade of life, when the lysosomal GL-3 accumulation becomes irreversible [7], [9] and patients are in need of dialysis or renal transplantation [9], [10]. The podocytes are the renal cells with the most abundant glycosphingolipid deposits [3], [8], [11], [12], [13] and this accumulation appears to be central for the development of nephropathy in Fabry disease [14], [15].

Lysosomal storage diseases in general [16], including Fabry disease, can now be treated with enzyme replacement therapy (ERT) [12], [17], [18], [19], [20], [21], [22], [23], [24], [25]. ERT reduces GL-3 deposits in podocytes [12], [22], [26] and can stabilize kidney function in patients with Fabry nephropathy [27]. Furthermore, for ERT to be maximally effective, it must be initiated early, before irreversible renal damage occurs [28], [29], [30].

Therapeutic effectiveness of α-Gal A is dependent on endocytosis by recognition of mannose 6-phosphate (M6P) residues on the enzyme by the widely distributed M6P/insulin-like growth II (IGF-II) receptor [31], [32], [33]. We previously showed that megalin, a multiligand receptor, binds α-Gal A and takes up α-Gal A in proximal tubule cells by receptor-mediated endocytosis [34]. However, the mechanism for the uptake in mouse podocytes in the same study was not investigated.

We have identified M6PR, but also megalin and sortilin as α-Gal A binding proteins important for α-Gal A accumulation in human podocytes by use of different biochemical and morphological approaches, including affinity chromatography, surface plasmon resonance analysis, laser microdissection, and immunocytochemistry to analyze human podocytes in culture and renal tissue from Fabry patients. On the basis of these data, we suggest a new and complex mechanism of α-Gal A cellular internalization in podocytes involving the sorting and endocytic receptors M6PR and sortilin and the high molecular weight endocytic receptor megalin.

## Results

### Podocytes take up recombinant α-Gal A

**Figure 1:**
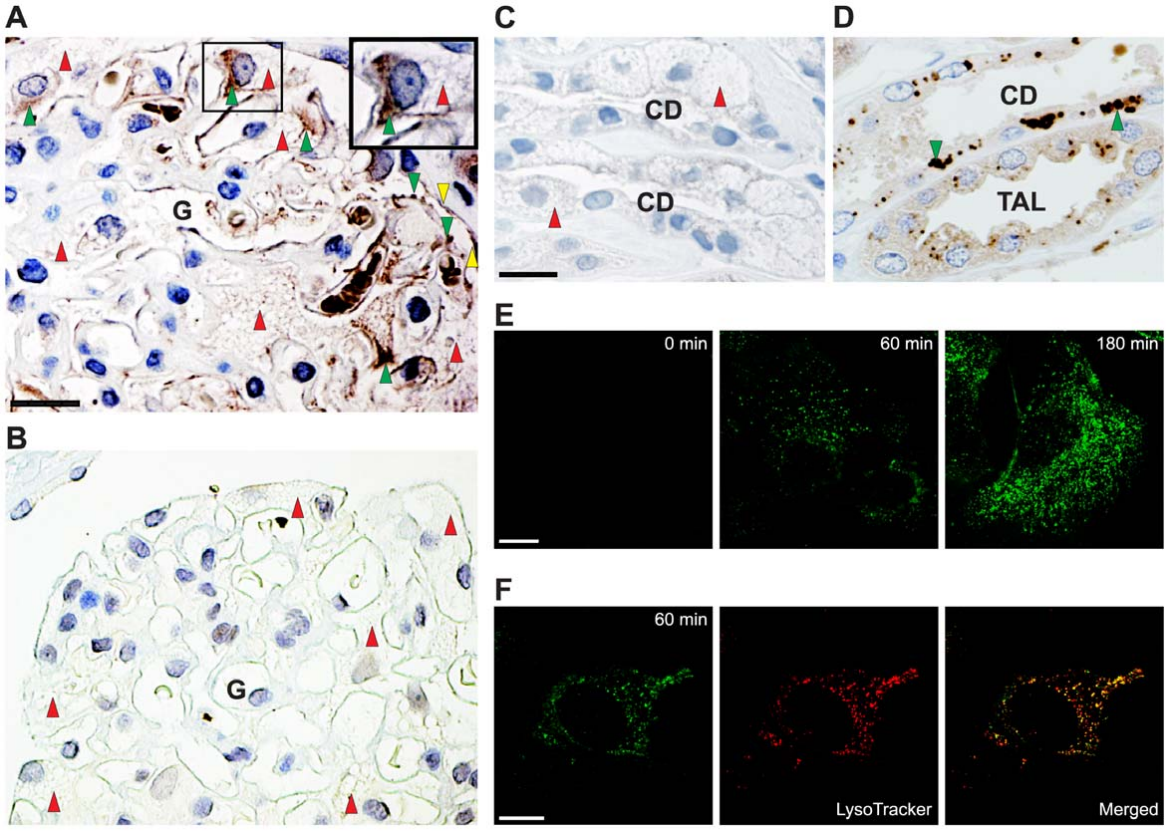
Uptake of recombinant α-Gal A by human podocytes. (A) Peroxidase immunohistochemistry for α-Gal A in a biopsy from a male Fabry patient using anti-human α-Gal A antibody. The patient was intravenously infused with α-Gal A 2 h before the biopsy was taken. Labeling of a human glomerulus (G) showing α-Gal A localization in the podocytes (indicated with green arrowheads) and GL-3 inclusions seen as vacuoles (indicated with red arrowheads). Staining is also seen in parietal epithelial cells (indicated with yellow arrows). Scale bar, 25 µm. A high-power view of a portion of the glomerulus (top-right) demonstrates the localization of infused recombinant α-Gal in the podocyte. (B) For comparison, no α-Gal A labeling is seen in the podocytes in a biopsy from an untreated male Fabry patient. (C) The treated male Fabry patient shows no detectable labeling of endogenous α-Gal A in the collecting ducts (CD). Red arrowheads indicate heavy GL-3 inclusions. Scale bar, 25 µm. (D) A normal individual shows labeling of endogenous α-Gal A (green arrowheads) in both thick ascending limbs of Henle (TAL) and in CD. Scale bar, 25 µm. (E) Immunofluorescent demonstration of Alexa-Fluor 488-labeled α-Gal A uptake in human podocytes as a function of time at 37°C. At the indicated times, the cells were fixed and analyzed by confocal microscopy. Scale bar, 10 µm. (F) For colocalization of α-Gal A (green) and lysosomes (red) a merged image is shown. The yellow color illustrates colocalization. Scale bar, 5 µm.

We have previously shown that endogenous α-Gal A is not detectable in normal human podocytes [34]. However in α-Gal A knockout mice, the enzyme was found in lysosomes of mouse podocytes 2 h after intravenous injection of recombinant α-Gal A [34]. To study if this was also the case in humans, we investigated a renal biopsy from a male Fabry patient infused with α-Gal A. Two hours after infusion of recombinant α-Gal A, the enzyme was localized in the podocytes (Figure 1A). For comparison, a renal biopsy from a male Fabry patient who was not infused with recombinant α-Gal A demonstrated no detectable α-Gal A in the podocytes (Figure 1B). Furthermore, the treated male Fabry patient showed no detectable endogenous α-Gal A in the collecting ducts (compare Figure 1C to Figure 1D from normal human kidney with normal expression).

To study in detail the podocyte uptake of α-Gal A, we used an already established human podocyte cell culture model [35]. Recombinant α-Gal A is also taken up by these conditionally immortalized podocytes showing time-dependent uptake (Figure 1E). The endocytosed enzyme was localized to the lysosomes as confirmed by colocalization of LysoTracker-Red with Alexa-Fluor-488 conjugated α-Gal A (Figure 1F).

### Isolation and identification of M6PR, megalin, and sortilin as α-Gal A-interacting proteins in podocytes

**Figure 2:**
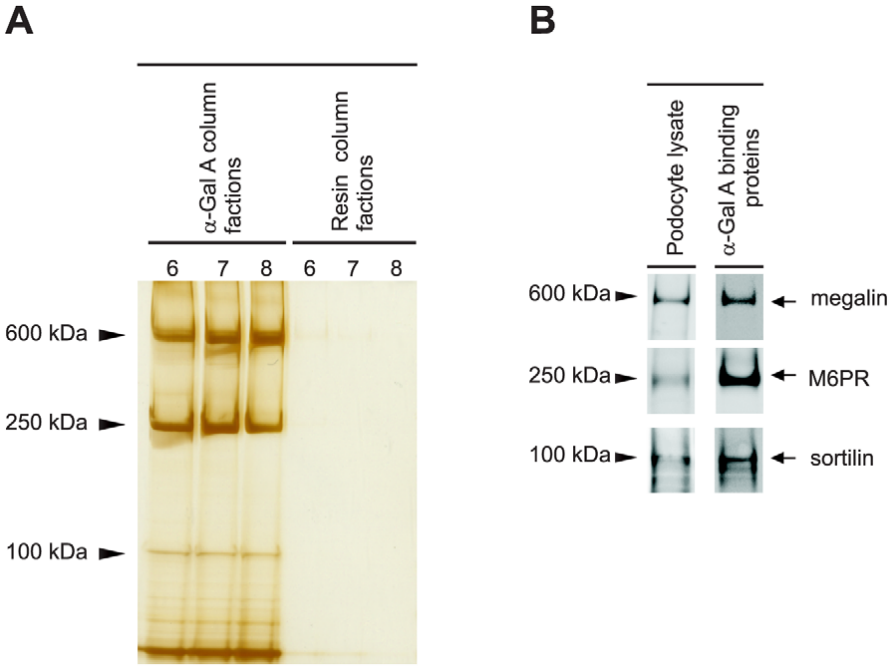
Megalin, M6PR and sortilin bind specifically to recombinant α-Gal A in podocytes. (A) Affinity chromatography: the arrowheads indicate the migration of the different α-Gal A binding proteins. (B) Western blot analysis demonstrated that the three bands were megalin, M6PR, and sortilin as indicated with arrows. Lysate from human podocyte culture was used as a positive control.

The receptor-mediated uptake of lysosomal hydrolases in podocytes has so far not been investigated. To identify receptors involved in the uptake of α-Gal A in podocytes, we employed an affinity-chromatography approach. A detergent-soluble extract of cultured podocytes was passed over recombinant α-Gal A affinity resin, and, following extensive washing, the resin bound proteins were eluted using pH 3 Glycine buffer. Fractions were collected and analyzed by SDS-PAGE and silver staining (Figure 2A). A sample of the same extract was also passed over a control resin to monitor nonspecific background binding of proteins to the resin. Comparison of the eluates from the two columns revealed that three protein bands with apparent masses of 600, 250 and 100 kDa were present in the fractions eluted from the α-Gal A resin but not the control resin (Figure 2A). To determine the identity of the proteins, the eluted fractions from the α-Gal A resin with the highest protein content were run on SDS-PAGE gel followed by immunoblotting. The proteins were identified as megalin, M6PR, and sortilin using the corresponding antibodies (Figure 2B).

### ^125^I-labeled α-Gal A uptake in human podocytes

**Figure 3:**
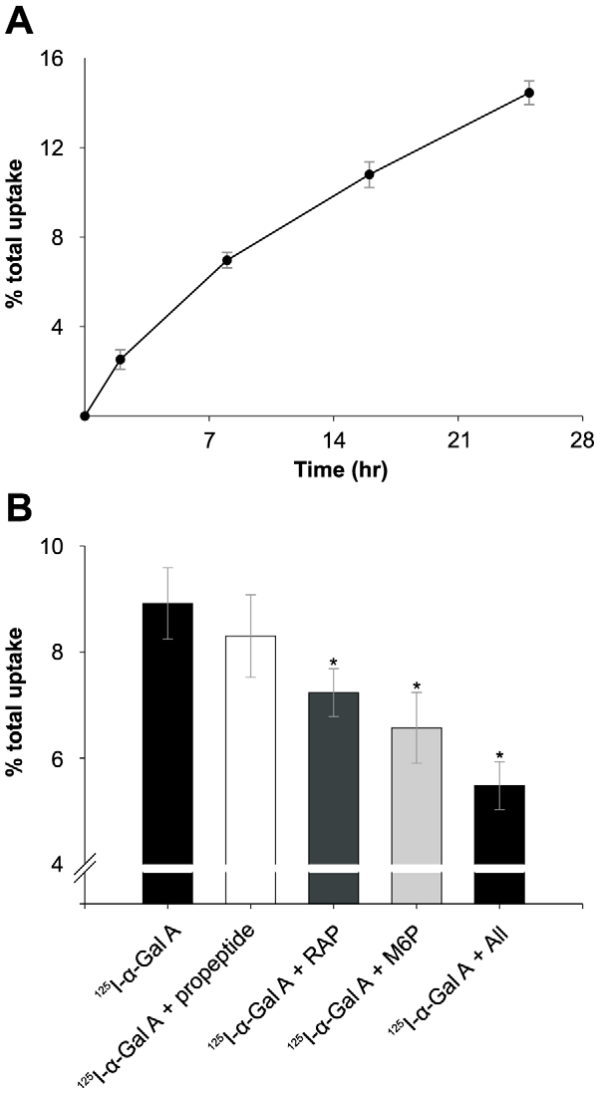
Uptake of ^125^I-labeled α-Gal A by human podocytes. (A) Human podocytes were incubated with ^125^I-α-Gal A for different times. (B) Podocytes incubated with ^125^I-α-Gal A for 12 h in the presence or absence of RAP, sortilin propeptide, M6P, and with all three ligands combined at 37°C. Uptake was assayed as described in the method section of the paper. Values are means of triplicate experiments with standard deviations (SD). The addition of sortilin propeptide shows no significant inhibition, however the addition of M6P, RAP or a combination of all three inhibitors show significant reductions (* indicate P<0.001) in the α-Gal A uptake after 12 h.

The endocytic activity of megalin, M6PR, and sortilin expressed by cultured human podocytes was investigated by their ability to mediate uptake of ^125^I-labeled α-Gal A (both cell association and degradation) (Figure 3). M6P, a ligand for M6PR, inhibited the α-Gal A uptake by approximately 26% after 12 h (P<0.001). RAP, a ligand for both megalin and sortilin, inhibited 19% (P<0.001), and sortilin propeptide, a specific sortilin ligand that is known to inhibit the binding/uptake of sortilin ligands [36], inhibited 7% after 12 h (not significant). Finally, the inhibition by all three ligands combined was tested, and the uptake of ^125^I-α-Gal A was inhibited 39% after 12 h (P<0.001).

### Sortilin, a new receptor for α-Gal A

**Figure 4:**
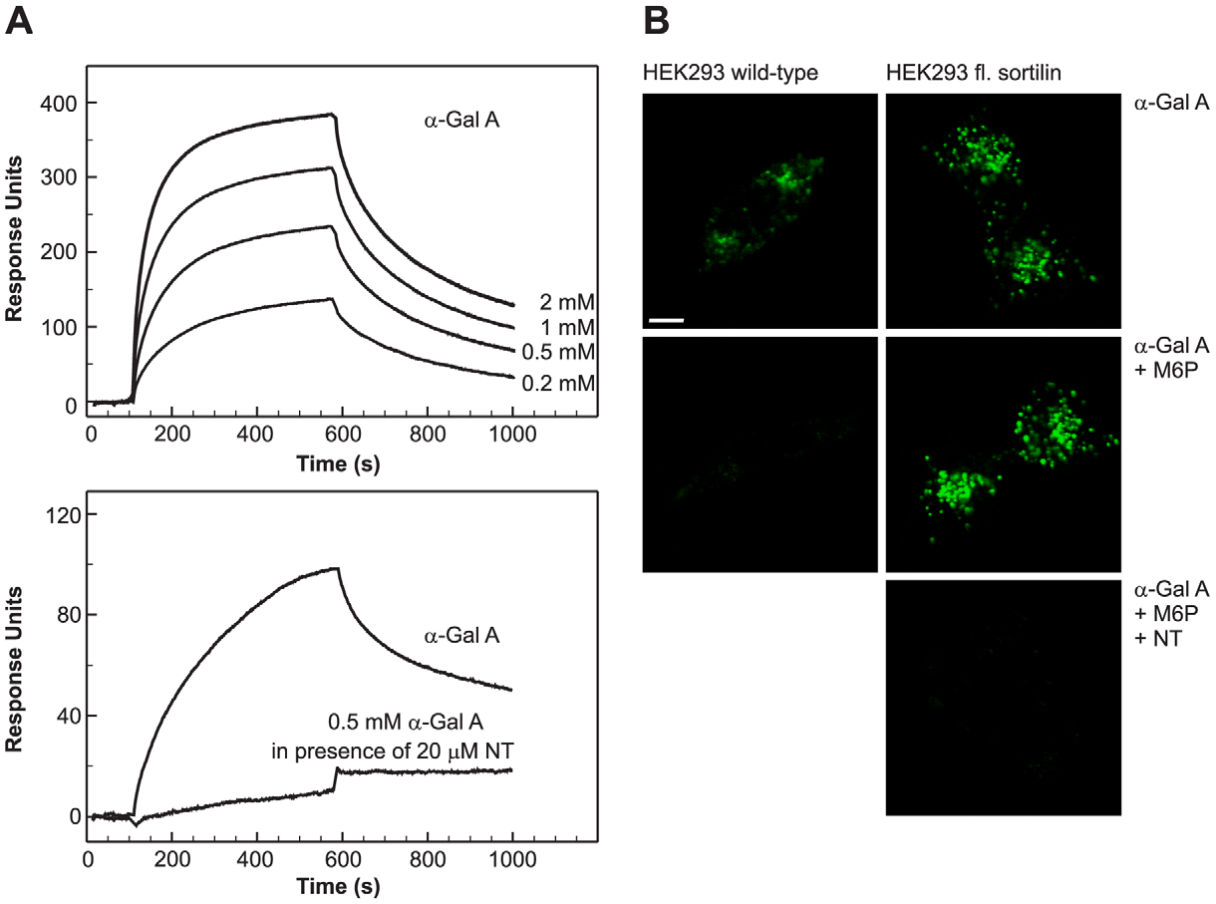
Binding and uptake of α-Gal A by sortilin. (A) SPR analysis of α-Gal A binding to purified human sortilin and inhibition of binding by NT. The lower binding curve was corrected for the binding of NT itself to sortilin. (B) Uptake of Alexa-Fluor 488-labeled α-Gal A by wild-type and full-length sortilin transfected HEK293 cells in the presence or absence of M6P or M6P and NT for 60 min at 37°C. At the given time, cells were fixed, and analyzed by confocal microscopy. Scale bar, 10 µm.

Surface plasmon resonance (SPR) analysis for megalin binding to α-Gal A was shown previously [34] with a K_d_ of 600 nM. Biacore analysis of α-Gal A binding to M6PR has already been shown in previous experiments [37]. We then focused on sortilin, SPR analysis showed binding of α-Gal A to purified and immobilized sortilin (Figure 4A). Using the BIAevaluation program, K_d_ was estimated to 400 nM. We next examined if neurotensin (NT), a neuropeptide with high affinity for sortilin [38], inhibited the binding of α-Gal A to sortilin. At 20 µM, NT markedly inhibited the binding of α-Gal A to sortilin (Figure 4A) indicating that NT and α-Gal A bind to sortilin at the same binding site as the sortilin propeptide. Other members of the Vps10p (vacuolar protein sorting 10 protein)-domain family [39], sorLA and sorCS3, did not show significant binding to α-Gal A (data not shown), and neither did the low density lipoprotein related protein (LRP). These results further substantiated that sortilin specifically binds α-Gal A.

Thus we had clear evidence that α-Gal A bound sortilin in order to demonstrate the ability of sortilin to take up α-Gal A. We developed a system in which sortilin is expressed in absence of megalin. We therefore used HEK293 cells stably transfected with sortilin to demonstrate α-Gal A binding to sortilin and uptake (Figure 4B). The transfectants expressed full-length sortilin in contrast to the wild-type cells. When cells were incubated in the presence of 5 mM M6P uptake by the M6PRs were totally inhibited in HEK293 wild-type but uptake was still present in the sortilin transfected HEK293 cells (Figure 4B). Next, we inhibited with NT and M6P, as shown in Figure 4B. NT at 20 µM essentially eliminated the binding and uptake of α-Gal A demonstrating the ability of sortilin to take up α-Gal A.

### Cultured human podocytes express megalin, sortilin and M6PR

**Figure 5:**
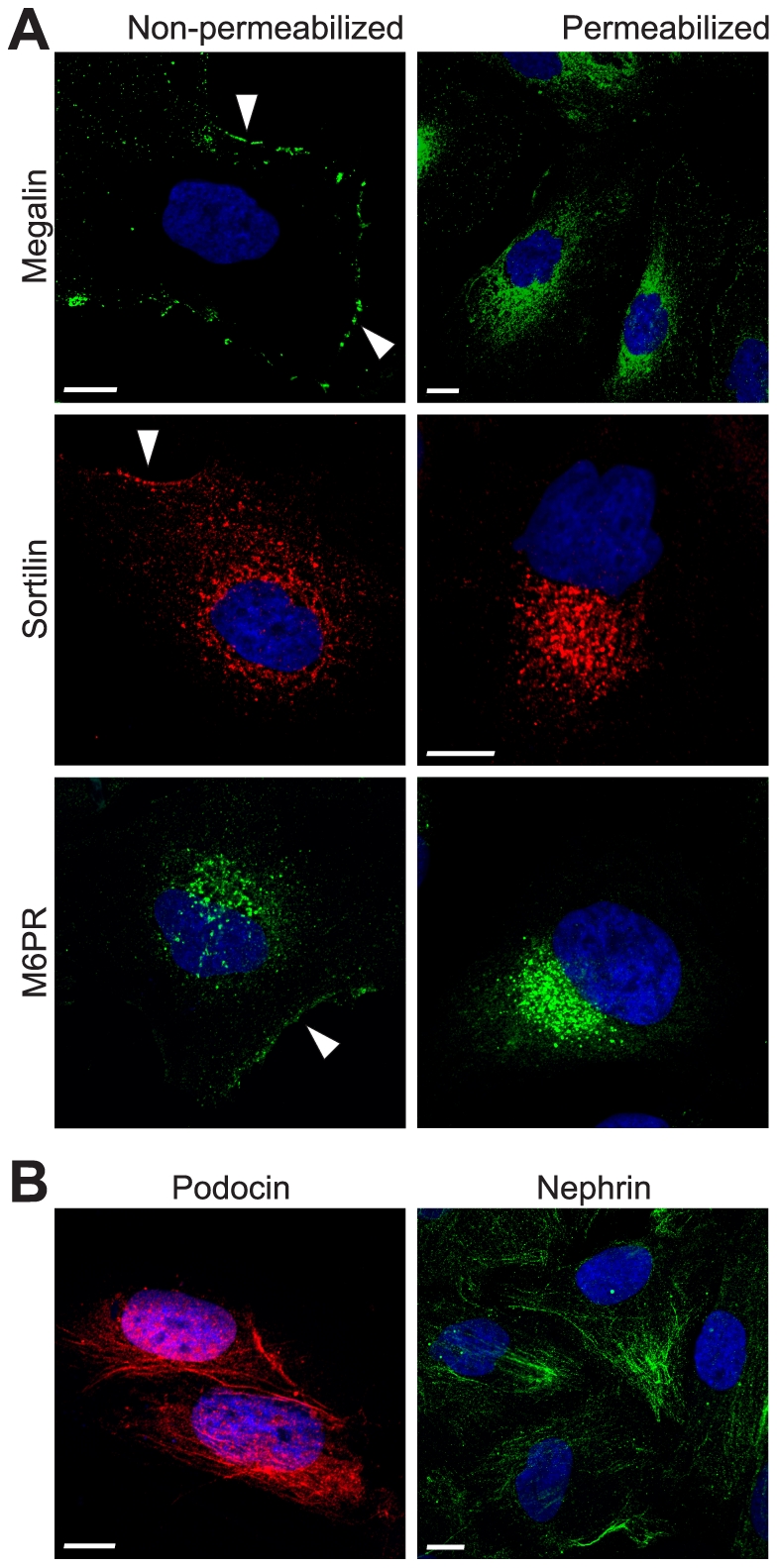
Megalin, sortilin, and M6PR are expressed in cultured podocytes. (A) Immunofluorescent demonstration of megalin, sortilin, and M6PR in cultured human podocytes and (B) specific podocyte-markers podocin and nephrin. Cells were fixed, stained with the appropriate antibodies and detected with Alexa-Fluor-conjugated secondary antibodies. Controls were incubated with serum and detected with same secondary antibodies (data not shown). Nuclei (blue) were stained with DAPI. Scale bars, 5 µm. White arrows indicate potential cell surface labeling of the different receptors in the human podocytes.

We next examined the surface expression of megalin, M6PR, and sortilin in cultured human podocytes by immunofluorescent studies under non-permeabilized conditions. It appears that megalin was predominantly expressed on the cell-surface, whereas M6PR and sortilin were mainly expressed intracellularly with some cell-surface expression (Figure 5). The expression of megalin, M6PR, and sortilin was also studied in permeabilized cells, where all three receptors demonstrated intracellular labeling (Figure 5). The phenotype of podocytes was characterized by using podocyte-specific antibodies to podocin and nephrin under permeabilized conditions (Figure 5). Controls incubated with serum or IgG fractions revealed no significant labeling.

### Expression of megalin, sortilin, and M6PR in human glomerular podocytes

**Figure 6:**
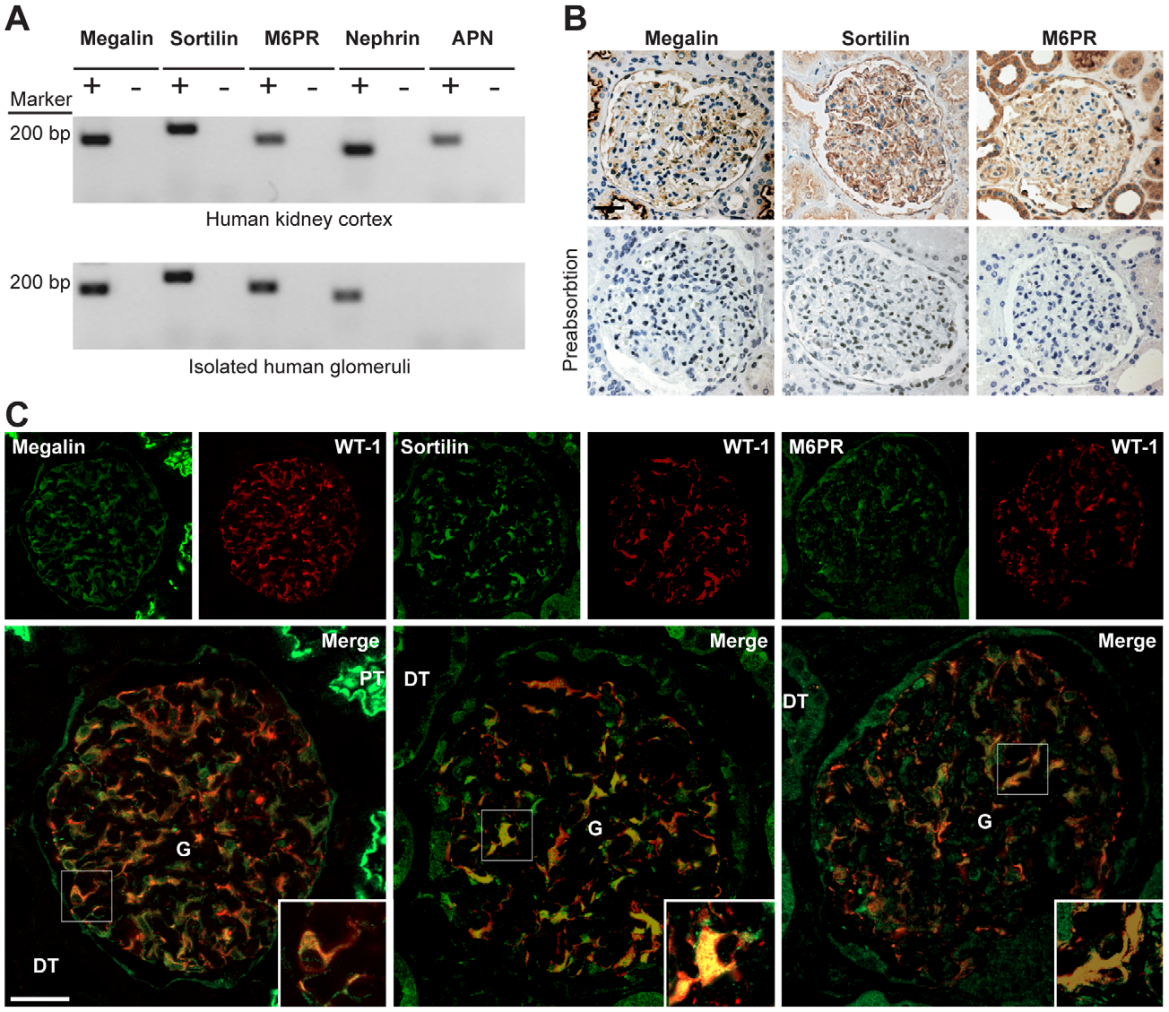
Expression of megalin, sortilin, and M6PR in human glomeruli. (A) Expression of megalin, M6PR, sortilin, podocin, nephrin, APN mRNAs by RT-PCR (+). Negative controls (−) run without RT enzyme. The marker used was 100 bp ladder. (B) Immunoperoxidase labeling of paraffin sections from a normal human kidney incubated with antibodies to megalin, M6PR, and sortilin show that the brush borders of proximal tubules (PT) are heavily stained. The distal tubules (DT) are unstained for megalin, but stained for sortilin and M6PR. The glomeruli (G) show staining for megalin, sortilin and M6PR. Preabsorption of the antibodies with the respective antigens show loss of immunoreactivity, demonstrating antibody specificity. Tubular and podocyte staining was blocked by preincubation. Controls that included only the secondary antibody showed no labeling (data not shown). Scale bar, 50 µm. (C) Dual immunofluorescence and confocal microscopy with monoclonal anti-WT1 antibody (red) and polyclonal antibodies (green) against megalin, sortilin, and M6PR. The respective merge images are shown in both low and high magnifications. Megalin, sortilin, and M6PR are localized in the glomerular podocytes and co-localizes with WT-1 as seen by the merged images indicated with white arrow-heads. High-power views of a portion of a glomerulus show merged images of the receptors and WT1 demonstrating that megalin, sortilin, and M6PR localize primarily to the podocyte cell bodies. Scale bar, 50 µm.

To study the expression of megalin, sortilin, and M6PR, we isolated human glomeruli by laser capture microdissection and evaluated if the receptors' mRNAs could be identified by RT-PCR. In order to avoid contamination with especially proximal tubule cells only the largest cross sections of the glomeruli from the 10 µm thick cryosections were chosen in order to be close to the equator, and furthermore the dissection was performed with safe distance to the glomerular parietal epithelium. To confirm, that the isolated glomeruli are not contaminated with proximal tubule, we demonstrate that the proximal tubule marker aminopeptidase N (APN) [40] is not present in the laser dissected glomeruli (Figure 6A). The expression of mRNA for megalin, M6PR, sortilin, podocin and nephrin is shown in the isolated human glomeruli and human kidney cortex in Figure 6A. No expression was observed in the negative controls.

To further confirm that the receptors were indeed expressed in the human glomerular podocytes, we performed immunohistochemistry studies on human kidney sections. We used three well characterized polyclonal antibodies to megalin, sortilin, and M6PR described in the literature. Immunoperoxidase staining using the three antibodies showed the presence of the receptors in the glomeruli and in the tubule segments (Figure 6B). This labeling was specific, as established by preabsorption of the three antibodies with their respective antigens (Figure 6B). We performed dual immunofluorescence using the three antibodies and a specific podocyte marker antibody against the Wilms' tumor 1 (WT1) protein to study the expression of the receptors in the podocytes of the human glomeruli. By immunolocalization, we detected megalin, sortilin, and M6PR localization to podocytes as demonstrated by colocalization with the podocyte cytoplasmic marker WT1 protein (Figure 6C). The receptors were present predominantly in podocyte cell bodies as seen on the merged high-power views of the glomeruli (Figure 6C).

## Discussion

In the present study, we have identified megalin, sortilin, and M6PR in human podocytes as endocytic receptors required for the delivery of recombinant α-Gal A to lysosomes. The M6PR mediated pathway has been very well characterized as the major route for the delivery of lysosomal enzymes; however, the mechanism for delivery of α-Gal A to lysosomes in human podocytes has been unclear until now. The heterogeneity of the uptake system of α-Gal A offers new perspectives to improve ERT for Fabry disease.

ERT given to Fabry patients has been shown to reduce GL-3 deposits in the podocytes [12], [22], [26], the principal affected cells in the progress of nephropathy in Fabry disease [3], [5], [6], [7]. The glomerular filtration barrier allows macromolecules such as albumin [41], transferrin, 80 kDa [42], and a pancreatic lipase, 110 kDa [43], to be filtered. Recently we have shown that there is a limited but significant glomerular filtration of recombinant α-Gal A in α-Gal A-deficient mice and Fabry patients [34]. These findings supported the hypothesis that ERT have an effect on podocytes, which has now been enforced by demonstration of uptake of recombinant α-Gal A by podocytes in a kidney from a Fabry disease patient (Figure 1A). The uptake of α-Gal A in the glomerular endothelial and the mesangial cells is currently under investigation and future studies will in detail reveal the uptake in these cells and the receptors involved.

We show here for the first time that megalin, sortilin, and M6PR are expressed in the human glomerular podocytes. Using laser capture microdissection to isolate human glomeruli, we show that mRNA transcripts of megalin, sortilin, and M6PR are expressed in human kidney cortex and in purified human glomeruli. Immunoperoxidase staining identified a specific glomerular staining with a pattern highly suggestive of podocyte labeling in the human kidney. Extraglomerular labeling was also present in tubule segments. Dual immunofluorescence study demonstrated that megalin, sortilin, and M6PR were all expressed in the podocytes as verified by perfect colocalization with WT1 and labeling was confirmed to the cell body of the podocytes. Furthermore, we also show that the expression of these receptor proteins can be detected by immunofluorescence and Western blot in human cultured podocytes.

Previous studies have described the presence of M6PR in the proximal tubule and glomeruli [44], [45]. Uptake of α-Gal A in podocytes was reduced by more than 25% with the addition of excess amounts of M6P. This was expected as the M6P residues on the recombinant α-Gal A are targeting the signal for the high-efficiency uptake by M6PR [32], [33]. M6PR is primarily (90–95%) localized in the intracellular compartments, particularly in the trans-Golgi network (TGN) and endosomes, with 5–10% of the receptor present on the cell surface [46]. The primary function of M6PR is to sort and transport M6P-bearing glycoproteins from TGN to endosomes/lysosomes [46]. In addition, M6PR can bind extracellular ligands and mediate endocytosis [47]. A small proportion of M6PR was localized on the cell surface in podocytes, and most of the M6PR was observed in intracellular compartments.

In our study we have shown that alternative M6PR-independent mechanisms exist for the uptake of α-Gal A. We expressed full-length sortilin in sortilin negative HEK293 cells. These transfected cells accumulated α-Gal A in intracellular compartments, and the uptake was inhibited with NT, a high affinity ligand for sortilin. These results demonstrated that sortilin is a multifunctional receptor that binds recombinant α-Gal A and, when expressed on the surface, mediates endocytosis. Sortilin is a multifunctional receptor belonging to the Vps10p-domain family receptors [38], [48] and strongly homologous to that of the M6PR [48]. Sortilin is like the M6PR mainly located in the Golgi compartment and vesicles [38], [48], but is also expressed on the cell surface. Sortilin has been shown to co-localize with the M6PR, both intracellularly and on the plasma membrane [49]. Thus, like the M6PR, sortilin has the potential of functioning both as a sorting receptor in the Golgi compartment and as a clearance receptor on the cell surface. Our studies demonstrated that sortilin like M6PR is located on the cell surface and in intracellular compartments in human podocytes. Uptake and degradation studies of ^125^I-labeled α-Gal A in human podocytes demonstrate that sortilin and M6PR participate in the endocytosis of α-Gal A. Our results show that sortilin could be a new receptor for α-Gal A endocytosis in podocytes.

Megalin, a multiligand endocytic receptor, binds many of the same ligands as sortilin, and is expressed in the renal tubular epithelium and in many other organs [50], [51]. Our results showed that megalin is present in human podocytes, and takes part in the uptake of α-Gal A. Megalin has previously not been described in human podocytes; however, it has been described and located in glomerular podocytes of rats [52], and also recently in cultured mouse podocytes [53]. The affinity of α-Gal A for binding to megalin is less than for sortilin [34]. Previous studies have shown that cubilin do not bind α-Gal A [34], and we did not find binding of α-Gal A to LRP (data not shown). These findings are consistent with our previous results showing that megalin is the receptor responsible for the proximal tubular accumulation of α-Gal A [34]. It has been shown that apolipoprotein B (apoB) and apoE rich lipoproteins are taken up by a receptor-mediated pathway in primary podocytes isolated from a human carcinoma kidney [54], and recently albumin was shown to be endocytosed by human podocytes in vitro and in vivo [55]. The uptake of these and other ligands could very well be facilitated by megalin, which is a receptor for apoB, apoE and albumin [50], [56], [57].

The contribution of each of the receptors in vivo is difficult to estimate based on our studies. RAP is a ligand for both megalin and sortilin and it binds to megalin with a higher affinity (8 nM) than for sortilin (78 nM) [58], [59]. Thus, the inhibition of ^125^I-labeled α-Gal A uptake with RAP may be due to the inhibition of both megalin and sortilin receptors.

The expression of megalin, sortilin, and M6PR in human podocytes is of general interest in the understanding of the rapidly progressing podocyte physiology. In addition, the expression of megalin may be of significance in immune-pathology. Megalin was initially discovered in 1982 as a pathogenic antigen of Heymann Nephritis in rats [60]. Our findings suggest that also in human, megalin may act as a pathogenic antigen for membranous glomerulonephritis. In the context of Fabry disease identification of two novel receptors on the podocyte opens new pathways of investigations to improve even further ERT.

In Fabry disease the effective targeting of recombinant α-Gal A depends on the M6P residues recognized by M6PR [31], [32], [33]. The limiting factor to this system includes the extent to which the recombinant enzyme is modified with M6P and the distribution of M6PR in different tissues. New approaches rely on the uptake mechanisms different from the M6P-based using protein-based mechanisms. Recently, a non-carbohydrate targeting platform for lysosomal proteins was evaluated [61]. The authors described that a fusion protein of β-glucuronidase with IGF-II, a high-affinity M6PR ligand, improved the targeting to podocytes in Mucopolysacccharidosis VII, another lysosomal disorder, where the podocyte is the most severely-affected kidney cell-type [62]. They also showed that α-Gal A modified with attachment of either apoE or IGF-II peptides increased the half-life by ∼20-fold in animal models, and that the in vitro uptake of these modified enzymes was not competed by M6P. Recent data have shown that fusion proteins between lysosomal enzymes and RAP were efficiently endocytosed by fibroblasts and other cell types from patients with lysosomal storage disorders. The uptake exceeded that of the phosphorylated enzyme and was presumably mediated by megalin and other members of low-density lipoprotein receptor (LDLR) family [63]. Since sortilin and megalin bind RAP, a fusion protein between α-Gal A and RAP could be used for a more efficient cellular delivery of α-Gal A using other pathways than M6PR. However, this will also target the other members of LDLR family, thus sortilin provides an alternative pathway using a fusion protein between α-Gal A and sortilin propeptide [36], and may deliver the therapeutic enzyme specifically via a sortilin mediated pathway.

In conclusion, we have identified M6PR, sortilin, and megalin as the receptors responsible for the lysosomal delivery of α-Gal A in human podocytes. Sortilin and megalin provide two M6P independent uptake systems for the delivery of the recombinant α-Gal A to the lysosomes in podocytes in Fabry disease patients. These findings are important steps in developing a rationale for ERT in Fabry disease.

## Materials and Methods

### Antibodies and proteins

Recombinant α-Gal A (Fabrazyme) and affinity-purified rabbit polyclonal anti-human α-Gal A were provided by Genzyme Corp. (Framingham, MA, USA). Sheep anti-rat megalin has been described earlier [64]. A specific polyclonal rabbit anti-human megalin was a kind gift from Dr. S. Moestrup, Aarhus University, Aarhus, DK. This antibody gave a specific band for megalin when tested by Western blot on human kidney homogenate. Recombinant receptor-associated protein (RAP) was provided by Novo Nordisk A/S (Bagsværd, DK) and Dr. S. Moestrup, Aarhus University, Aarhus, DK. Affinity purified rabbit polyclonal anti-sortilin was generated against the ectodomain of human sortilin, as previously described [36]. Polyclonal rabbit anti-sortilin (ab16640) and sortilin peptide (ab16686) were purchased from Abcam. Recombinant GST-sortilin propeptide were expressed in *Escherichia coli* and purified as previously described [36]. A specific affinity purified rabbit polyclonal anti-IGF-II/M6PR was generated against the full-length bovine IGF-II/M6PR as previously described [65]. Polyclonal rabbit anti-rat IGF-II/M6PR 3637 was a kind gift from Dr. W. Kiess, Hospital for Children and Adolescence, University of Leipzig, Leipzig, DE. Recombinant human IGF-II receptor (2447-GR) was purchased from R & D systems (Minneapolis, MN, USA). The mannose 6-phosphate (M6P; M3655), neurotensin (NT; N6383), affinity purified rabbit polyclonal anti-podocin (P0372) were purchased from Sigma Aldrich (Saint Louis, MO, USA). Monoclonal mouse anti-nephrin IgG1 was a kind gift from Dr. K. Tryggvason, Karolinska Institute, Stockholm, SE. Monoclonal mouse anti-human Wilms' tumor 1 (WT1; clone 6F-H2) protein and peroxidase-conjugated secondary antibodies were purchased from DAKO A/S (Glostrup, DK). Fluorescence-conjugated secondary antibodies were purchased from Molecular Probes (Eugene, OR, USA). Controls for unspecific binding were performed with nonspecific rabbit, mouse, or sheep IgG from DAKO.

### Cells

The human podocyte cell line conditionally immortalized by introducing temperature-sensitive SV40-T antigen by transfection has previously been characterized in detail [35]. The podocyte cell line (passages 12 to 25) was maintained in RPMI 1640 (R-8758) medium supplemented with insulin, transferrin, selenite (ITS; I-3146), 10% FBS (F7524) all from Sigma Aldrich, at 33°C in 5% CO_2_. Podocyte differentiation was induced under nonpermissive conditions by thermo shifting the cells to 37°C for 14 days. HEK 293 cells were obtained from Invitrogen (Carlsbad, CA, USA) and maintained in DMEM (LONZA, BE) supplemented with 10% FBS (GIBCO, Paisley, UK), at 37°C in 5% CO_2_. The human cDNA construct encoding full-length sortilin [66] was expressed using the mammalian expression vector pcDNA3.1/zeo (Invitrogen, Groningen, NL). Cells were transfected with pcDNA3.1/zeo using FUGENE 6 (Roche, CH), and a stably transfected clone was selected in medium containing 150 µg/ml zeocin. All cell culture media were supplemented with 100 U/ml penicillin, and 100 µg/ml streptomycin (LONZA). The media was renewed every second day, and cells were split at confluence approximately once a week using a trypsin-EDTA solution (LONZA). Experiments were carried out with confluent monolayers of cells cultured in 24-well plates (Nagle Nunc International, Hereford, UK) with or without cover slips for uptake studies, and in 75 cm^2^ culture flasks (Corning Incorporated, Corning, NY, USA) for the affinity purification experiment. Podocytes were only used after they were differentiated under non-permissive conditions for 14 days at 37°C.

### Biopsy preparation

Renal biopsies were obtained from A) a kidney from a male Fabry patient, 37 years of age, 2 h after enzyme replacement infusion, 0.2 mg/kg body wt recombinant α-Gal A and B) an untreated male Fabry patient, 38 years of age. The biopsies were prepared for paraffin-embedding by routine methodology. Ethical approval for the human studies was granted by the Copenhagen Local Research Ethics Committee and informed consent was obtained from the patient.

### Immunofluorescence microscopy of cell cultures

#### Uptake of Alexa Fluor 488-labeled α-Gal A in cultured human podocytes

Recombinant α-Gal A was labeled with Alexa Fluor 488 according to the instructions of the manufacturer (Molecular Probes). Podocytes, parental and full-length sortilin HEK293 cells were incubated with Alexa Fluor 488-labeled α-Gal A at 37°C at indicated times with or without inhibitors, and fixed with 4% paraformaldehyde for 10 min at room temperature. LysoTracker Red DND-99 (L-7528; Molecular Probes) was used as described by the manufacturer. Cells were counterstained with LysoTracker Red for 15 min before fixation.

#### Localization of proteins in podocytes

Immunofluorescence on human podocytes was performed as described previously [35] at room temperature. In brief, cover slips were fixed with 2% paraformaldehyde, 4% sucrose in PBS for 10 min and permeabilized with 0.3% Triton X-100 (Sigma Aldrich) in PBS for 10 min. Nonspecific binding sites were blocked with 4% FBS+0.1% Tween 20 (Sigma Aldrich) in PBS for 60 min. Primary and secondary antibodies were applied at the appropriate dilutions according to standard techniques.

#### Cell surface localization of proteins in podocytes

For surface labeling of proteins, cover slips were fixed with 2% paraformaldehyde, 4% sucrose in phosphate-buffered saline and incubated with primary antibodies over night at 4°C. After this, they were incubated with Alexa Fluor conjugated antibodies for 1 h at room temperature, and nuclei-stained with DAPI (Molecular Probes) according to the directions from manufacturer.

All cover slips were mounted on glass slides in DAKO Fluorescent Medium. Staining was analyzed by confocal laser scanning microscope (LSM510; Carl Zeiss, DE). Images were acquired using the software from the manufacturer and processed with Adobe Photoshop CS3 software. Controls for nonspecific binding were performed with nonspecific serum and IgG fractions.

### Protein extraction

Cell proteins were extracted by addition of modified RIPA buffer (R-0278; Sigma Aldrich) containing 50 mmol/L Tris-HCL, 150 mmol/L NaCl, 1% Triton X-100, 1 mmol/L ethylenediaminetetraacetic acid, 1 mmol/L phenylmethyl sulfonyl fluoride, 1 µg/mL aprotinin, 1 µg/mL leupeptin, 1 µg/mL pepstatin, 1 mmol/L Na vanadate; and complete protease inhibitor (Roche Diagnostics, Mannheim, DE; 1 tablet per 50 ml of solution) at 4°C. The suspension was centrifuged at 14000×g for 15 min at 4°C, and the supernatant containing cellular protein was collected. Total protein was quantified using Pierce BCA Protein Assay kit (Pierce, Rockford, IL, USA) according to the manufacturer.

### Affinity purification of α-Gal A binding proteins

Purified recombinant α-Gal A covalently coupled using the AminoLink Plus Immobilization Kit (Pierce) following the guidelines from the manufacturer. Affinity purification was done following the Pierce instructions. Briefly, differentiated podocytes were lysed with RIPA buffer as described before. The cell lysate was diluted in PBS binding buffer (pH 7.4) at a ratio of at least 1∶4 and recirculated over the column three times at room temperature. The column was extensively washed in wash solution (1 M NaCl, 0.05% NaN_3_) followed by elution of bound proteins with 0.2 M glycine·HCl buffer (pH 2.5). Fractions (300 µl) were collected, subjected to SDS-PAGE, and stained by silver staining using SilverSNAP Stain Kit II (Pierce). Additional gels were blotted onto PVDF membranes for further protein identification by Western blotting.

### SDS-PAGE and Western blotting

Eluted α-Gal A binding protein fraction with the highest protein content and podocyte lysates were loaded on the SDS-PAGE gels. The samples were mixed with NuPAGE LDS sample buffer (Invitrogen, Carlsbad, CA) with 2.5% SDS, and the proteins were separated by SDS-PAGE and electrophoretically transferred to PVDF membranes (Millipore Corporation; Bedford, MA, USA) for Western blot. Blots were blocked with 5% milk in PBS-T (80 mM Na_2_HPO_4_, 20 mM NaH_2_PO_4_, 100 mM NaCl, and 0.1% Tween 20 [pH 7.5]) for 1 h and incubated overnight at 4°C with primary antibody in PBS-T with 1% BSA. After washing in PBS-T, the blots were incubated for 1 h with horseradish peroxidase-conjugated secondary antibody (DAKO). After a final wash, antibody binding was visualized using ECL system (Amersham International, Bucks, UK).

### Surface plasmon resonance analysis

For the surface plasmon resonance (SPR) analyses, BIAcore sensor chips (type CM5; Biacore, Uppsala, SE) were activated with a 1∶1 mixture of 0.2 M *N*-ethyl-N′-(3-dimethylaminopropyl) carbodiimide and 0.05 M *N*-hydroxysuccinimide in water according to the manufacturer. Sortilin was immobilized as previously described [36]. The SPR signal from immobilized sortilin generated BIAcore resonance units (RU) that were equivalent to 66 fmol/mm^2^. The flow cells were regenerated with 20 µl of 1.5 M glycine-HCl (pH 3.0). The flow buffer was 10 mM HEPES, 150 mM NaCl, 1.5 mM CaCl_2_, and 1 mM EGTA (pH 7.4). The binding data was analyzed using the BIAevaluation program. The number of ligands bound per immobilized receptor was estimated by dividing the ratio RU ligand/mass ligand with RU receptor/mass receptor. The K_d_ for binding was estimated using a BIA evaluation program.

### ^125^I-labeled α-Gal A uptake by podocytes

Lysosomal α-Gal A was iodinated with the use of the chloramine-T method [67] to a specific activity of approximately 217,000 cpm/µg protein. Human podocytes were incubated with 10 nM ^125^I-α-Gal A in the presence or absence of 1 µM RAP, 10 µM M6P, 1 µM sortilin propeptide, and all three ligands at 37°C in 5% CO2/95% air for 4, 8, and 12 h. Incubation with ^125^I-α-Gal A was carried out in serum-free medium (RPMI1640) containing 0.1% BSA (Sigma Aldrich), and stopped by aspiration of the medium. Cells were washed in PBS and collected by trypsinization with 150 µl of Trypsin-EDTA. The samples were counted in a γ-counter (Cobra 5003, Packard, Meriden, CT, USA). Degradation was measured by precipitation of the medium in 10% TCA and the TCA-soluble fraction, defined as the degraded fraction. To correct for liberation *per se* of iodine from ^125^I-α-Gal A during the experiment, the medium in control wells was incubated without cells. Degradation was calculated as the TCA-soluble fraction in the incubation medium minus the TCA-soluble fraction in the medium from control wells. Cell-associated ^125^I-α-Gal A was measured by counting the cells after trypsinization. Total uptake was defined as the sum of degraded and cell-associated fraction divided by the total amount of tracer added in each well. The medium from control wells contained a TCA-soluble fraction of approximately 10%.

### Tissue Preparation and LCM

Human kidneys were obtained from renal carcinoma patients. Ethical approval for the human studies was granted by the Local Research Ethics Committee and informed consent was obtained from the patients. Immediately after surgical removal, carcinoma free parts of the kidneys were excised, embedded in Tissue-Tek OCT Compound, and immediately snap-frozen, placed on dry ice and stored at −80°C until use. Tissues were cryosectioned into 10 µm thick sections at −20°C using a cryostat. Cryosections were then attached to RNase-free glass slides (Arcturus, Mountain View, CA, USA). The slides were processed with an RNase-free technique. The slides were thawed at room temperature for 30 sec and then dehydrated in 75% ethanol for 1 min, 95% ethanol for 1 min, and 100% ethanol for 1 min. Finally, sections were incubated in xylene for 5 min and air dried for 5 min.

Immediately after dehydration LCM was performed using the automated VERITAS™ Microdissection Instrument (Arcturus). The laser beams were adjusted to cut and capture the visualized glomeruli. Dissection was performed at room temperature and was limited to 30 min to prevent RNA degradation. CapSure HS LCM caps (Arcturus) were used to capture the glomeruli, ensuring precise capture and preventing contamination from the surrounding tissue while microdissecting. Furthermore, CapSure HS LCM caps together with ExtracSure devices (Arcturus) enabled the highly sensitive extraction of glomeruli. For RNA extraction, a total of 200 glomeruli were captured.

### RNA Isolation and RT-PCR

Total human glomerular- and cortex RNA were extracted using the PicoPure RNA Isolation Kit (Arcturus) as described by the manufacturer. DNase treatment was performed on PicoPure column with a RNase-free DNase set (Qiagen, Hilden, DE). Total RNA was estimated using a NanoDrop 2000 (Thermo Scientific, Wilmington, DE, USA), showing that each sample yielded 3–8 ng/µl.

Reverse transcription was performed for 60 minutes at 42°C in a volume of 20 µl using Sensiscript RT (Qiagen) and respective buffer. This was performed in the presence of 0.5 mM deoxyribonucleoside triphosphates (dATP, dTTP, dGTP, dCTP) (Qiagen), 1 µM of a 16mer d(T) oligonucleotide primer (Perkin Elmer, USA), 0.5 unit/µl RNase inhibitor (Perkin Elmer), 1 µl Sensiscript RT and purified RNA (25 ng). A reaction without RT enzyme was made in parallel with purified RNA to ascertain that the amplification occurred on RNA and not DNA.

PCR was performed in a volume of 10 µl using a HotStarTaq Master Mix Kit (Qiagen) following the protocol from manufacturer. This was performed with 5 µl HotStarTaq Master Mix, 2.5 µl cresol, 5 pmol of sense and antisense primers (Table 1) and 1 µl of the RT-product. The PCR program was as follows: denaturation 15 min 94°C and 35 cycles of: 1 min 94°C, 30 sec 58°C, 1 min 72°C. After cycling the reaction ended with 10 min at 72°C. After the PCR reaction the products were visualized by running a 1.5% agarose gel stained with ethidium bromide (0.5 µg/ml) and developed using Fluor-S™ MultiImager (BIO RAD, USA).

**Table 1:** Primers for RT-PCR.

Transcript	GenBank accession #	Primer pair	Product size *bp*
Megalin	NM_004525.2	5′-AGCCTCTGGAGTTGGACAGA-3′ (forward)5′-ACAGTGCGGTTAGACCCATC-3′ (reverse)	189
M6PR	NM_000876.2	5′-AGTGGAAGGGGACAACTGTG-3′ (forward)5′-ACATGAGGAGACCACCTTGG-3′ (reverse)	184
Sortilin	NM_002959.4	5′-TCCTGGGTTGGAGATAGCAC-3′ (forward)5′-TTCCTCCAGACACCTCTGCT-3′ (reverse)	232
Podocin	NM_014625.1	5′-TGGGGAATCAAAGTGGAGAG-3′ (forward)5′-GAATCTCAGCTGCCATCCTC-3′ (reverse)	169
Nephrin	NM_004646.1	5′-GACCCAGCTTCCCATCACTA-3′ (forward)5′-GCATTGGAGAGGAGCAGAAG-3′ (reverse)	150
Aminopeptidase N	NM_001150.2	5′-CAGGGGCCTGTACGTTTTTA-3′ (forward)5′-CCACCAGCTCAGTCTTGTCA-3′ (reverse)	179

### Immunohistochemistry

Renal sections were obtained from paraffin-embedded Fabry disease kidneys or normal human kidneys (autopsy materials). For light microscope immunohistochemistry, 2-µm tissue paraffin sections were cut on a Leica RM 2165 microtome (Leica, Wetzlar, Germany). Sections were dried at 60°C in an oven for 1 h, placed in xylene overnight, rehydrated in graded alcohols, heated in TEG buffer (Tris-EGTA buffer, pH 9) at approximately 100°C in a microwave oven for 20 min, cooled at room temperature for 30 min, incubated for 30 min in 50 nM NH_4_Cl in 0.01 M PBS. Sections were permeabilized with 0.05% saponin (1% BSA, 0.2% gelatine, 0.05% saponin in 0.01 M PBS) and blocked for endogenous peroxidase before incubation with the primary antibody. Sections were incubated with a primary antibody in 0.01 M PBS, 0.1% BSA and 0.02 M NaN_3_, followed by incubation with HRP-conjugated secondary antibody. For the preabsorption experiments, primary polyclonal rabbit anti-megalin, anti-sortilin and anti-M6PR antibodies were incubated with affinity purified megalin, sortilin, and M6PR respectively for 2 h at room temperature before incubating the preincubated antibody and antigen mixture on the tissue-slide. Megalin was purified from human cortical membranes on RAP affinity column, using the AminoLink Plus Immobilization Kit and human cortical membranes were obtained from human kidneys and prepared as previously described [68]. Sortilin and M6PR were purchased from Abcam and R&D systems, respectively. Sections were counterstained with Meier's haematoxylin stain and peroxidase labeling was visualized by incubation with diaminobenzidine and H_2_O_2_ for 10 min.

For dual immunofluorescence labeling mouse anti-WT1 and rabbit anti-megalin (antiserum), anti-sortilin (immunopurified polyclonal antibody to human sortilin), or anti-M6PR (immunopurified polyclonal antibody to bovine M6PR) antibodies were applied followed by Alexa Fluor conjugated anti-mouse and anti-rabbit IgG. All incubations with primary antibodies were done overnight at 4°C and secondary incubations were done at room temperature for 1 hour. Sections were subsequently examined in a Leica DMR microscope equipped with a Leica DFC320 camera or a Zeiss LSM510 Meta laser confocal microscope. Images were transferred by a Leica TFC Twain 6.1.0 program and processed using Adobe Photoshop 8.0. Negative controls were performed by replacing the primary antibody with the normal serum of the species in which the primary antibody was raised or without the primary antibody.

### Statistical analysis

The results of ^125^I- α-Gal A uptake experiments are presented as means ± SD, and the t test was used to test for significant differences. P values<0.05 were considered significant.

## References

[1] Brady RO, Gal AE, Bradley RM, Martensson E, Warshaw AL (1967). Enzymatic defect in Fabry's disease. Ceramidetrihexosidase deficiency.. N Engl J Med.

[2] Kint JA (1970). Fabry's disease: alpha-galactosidase deficiency.. Science.

[3] Desnick RJ, Ioannou YA, Eng CM, Scriver CRBA, Sly WS, Valle D (2006). α-Galactosidase A deficiency: Fabry disease.. The Metabolic and Molecular Bases of Inherited Disease. 8 ed.

[4] Mehta A, Ricci R, Widmer U, Dehout F, Garcia de Lorenzo A (2004). Fabry disease defined: baseline clinical manifestations of 366 patients in the Fabry Outcome Survey.. Eur J Clin Invest.

[5] Colley JR, Miller DL, Hutt MS, Wallace HJ, De Wardener HE (1958). The renal lesion in angiokeratoma corporis diffusum.. Br Med J.

[6] Meroni M, Sessa A, Battini G, Tazzari S, Torri Tarelli L (1997). Kidney involvement in Anderson-Fabry disease.. Contrib Nephrol.

[7] Branton MH, Schiffmann R, Sabnis SG, Murray GJ, Quirk JM (2002). Natural history of Fabry renal disease: influence of alpha-galactosidase A activity and genetic mutations on clinical course.. Medicine (Baltimore).

[8] Najafian B, Svarstad E, Bostad L, Gubler MC, Tondel C (2011). Progressive podocyte injury and globotriaosylceramide (GL-3) accumulation in young patients with Fabry disease.. Kidney Int.

[9] Thadhani R, Wolf M, West ML, Tonelli M, Ruthazer R (2002). Patients with Fabry disease on dialysis in the United States.. Kidney Int.

[10] Ojo A, Meier-Kriesche HU, Friedman G, Hanson J, Cibrik D (2000). Excellent outcome of renal transplantation in patients with Fabry's disease.. Transplantation.

[11] Gubler MC, Lenoir G, Grunfeld JP, Ulmann A, Droz D (1978). Early renal changes in hemizygous and heterozygous patients with Fabry's disease.. Kidney Int.

[12] Thurberg BL, Rennke H, Colvin RB, Dikman S, Gordon RE (2002). Globotriaosylceramide accumulation in the Fabry kidney is cleared from multiple cell types after enzyme replacement therapy.. Kidney Int.

[13] Tondel C, Bostad L, Hirth A, Svarstad E (2008). Renal biopsy findings in children and adolescents with Fabry disease and minimal albuminuria.. Am J Kidney Dis.

[14] Alroy J, Sabnis S, Kopp JB (2002). Renal pathology in Fabry disease.. J Am Soc Nephrol.

[15] Meehan SM, Junsanto T, Rydel JJ, Desnick RJ (2004). Fabry disease: renal involvement limited to podocyte pathology and proteinuria in a septuagenarian cardiac variant. Pathologic and therapeutic implications.. Am J Kidney Dis.

[16] Hers HG (1965). Inborn Lysosomal Diseases.. Gastroenterology.

[17] Schiffmann R, Murray GJ, Treco D, Daniel P, Sellos-Moura M (2000). Infusion of alpha-galactosidase A reduces tissue globotriaosylceramide storage in patients with Fabry disease.. Proc Natl Acad Sci U S A.

[18] Schiffmann R, Kopp JB, Austin HA, Sabnis S, Moore DF (2001). Enzyme replacement therapy in Fabry disease: a randomized controlled trial.. JAMA.

[19] Eng CM, Banikazemi M, Gordon RE, Goldman M, Phelps R (2001). A phase 1/2 clinical trial of enzyme replacement in fabry disease: pharmacokinetic, substrate clearance, and safety studies.. Am J Hum Genet.

[20] Schiffmann R, Ries M, Timmons M, Flaherty JT, Brady RO (2006). Long-term therapy with agalsidase alfa for Fabry disease: safety and effects on renal function in a home infusion setting.. Nephrol Dial Transplant.

[21] Schiffmann R, Askari H, Timmons M, Robinson C, Benko W (2007). Weekly enzyme replacement therapy may slow decline of renal function in patients with Fabry disease who are on long-term biweekly dosing.. J Am Soc Nephrol.

[22] Germain DP, Waldek S, Banikazemi M, Bushinsky DA, Charrow J (2007). Sustained, long-term renal stabilization after 54 months of agalsidase beta therapy in patients with Fabry disease.. J Am Soc Nephrol.

[23] Banikazemi M, Bultas J, Waldek S, Wilcox WR, Whitley CB (2007). Agalsidase-beta therapy for advanced Fabry disease: a randomized trial.. Ann Intern Med.

[24] West M, Nicholls K, Mehta A, Clarke JT, Steiner R (2009). Agalsidase alfa and kidney dysfunction in Fabry disease.. J Am Soc Nephrol.

[25] Mehta A, Beck M, Elliott P, Giugliani R, Linhart A (2009). Enzyme replacement therapy with agalsidase alfa in patients with Fabry's disease: an analysis of registry data.. Lancet.

[26] Eng CM, Guffon N, Wilcox WR, Germain DP, Lee P (2001). Safety and efficacy of recombinant human alpha-galactosidase A–replacement therapy in Fabry's disease.. N Engl J Med.

[27] Fervenza FC, Torra R, Warnock DG (2008). Safety and efficacy of enzyme replacement therapy in the nephropathy of Fabry disease.. Biologics.

[28] Breunig F, Weidemann F, Strotmann J, Knoll A, Wanner C (2006). Clinical benefit of enzyme replacement therapy in Fabry disease.. Kidney Int.

[29] Vedder AC, Linthorst GE, Houge G, Groener JE, Ormel EE (2007). Treatment of Fabry disease: outcome of a comparative trial with agalsidase alfa or beta at a dose of 0.2 mg/kg.. PloS ONE.

[30] Oqvist B, Brenner BM, Oliveira JP, Ortiz A, Schaefer R (2009). Nephropathy in Fabry disease: the importance of early diagnosis and testing in high-risk populations.. Nephrol Dial Transplant.

[31] Dahms NM, Lobel P, Kornfeld S (1989). Mannose 6-phosphate receptors and lysosomal enzyme targeting.. J Biol Chem.

[32] Sando GN, Neufeld EF (1977). Recognition and receptor-mediated uptake of a lysosomal enzyme, alpha-l-iduronidase, by cultured human fibroblasts.. Cell.

[33] Kaplan A, Achord DT, Sly WS (1977). Phosphohexosyl components of a lysosomal enzyme are recognized by pinocytosis receptors on human fibroblasts.. Proc Natl Acad Sci U S A.

[34] Christensen EI, Zhou Q, Sorensen SS, Rasmussen AK, Jacobsen C (2007). Distribution of alpha-galactosidase A in normal human kidney and renal accumulation and distribution of recombinant alpha-galactosidase A in Fabry mice.. J Am Soc Nephrol.

[35] Saleem MA, O'Hare MJ, Reiser J, Coward RJ, Inward CD (2002). A conditionally immortalized human podocyte cell line demonstrating nephrin and podocin expression.. J Am Soc Nephrol.

[36] Munck Petersen C, Nielsen MS, Jacobsen C, Tauris J, Jacobsen L (1999). Propeptide cleavage conditions sortilin/neurotensin receptor-3 for ligand binding.. EMBO J.

[37] Lee K, Jin X, Zhang K, Copertino L, Andrews L (2003). A biochemical and pharmacological comparison of enzyme replacement therapies for the glycolipid storage disorder Fabry disease.. Glycobiology.

[38] Mazella J, Zsurger N, Navarro V, Chabry J, Kaghad M (1998). The 100-kDa neurotensin receptor is gp95/sortilin, a non-G-protein-coupled receptor.. J Biol Chem.

[39] Willnow TE, Petersen CM, Nykjaer A (2008). VPS10P-domain receptors - regulators of neuronal viability and function.. Nat Rev Neurosci.

[40] Rahmoune H, Thompson PW, Ward JM, Smith CD, Hong G (2005). Glucose transporters in human renal proximal tubular cells isolated from the urine of patients with non-insulin-dependent diabetes.. Diabetes.

[41] Brenner BM, Baylis C, Deen WM (1976). Transport of molecules across renal glomerular capillaries.. Physiol Rev.

[42] Kozyraki R, Fyfe J, Verroust PJ, Jacobsen C, Dautry-Varsat A (2001). Megalin-dependent cubilin-mediated endocytosis is a major pathway for the apical uptake of transferrin in polarized epithelia.. Proc Natl Acad Sci U S A.

[43] Comte B, Franceschi C, Sadoulet MO, Silvy F, Lafitte D (2006). Detection of bile salt-dependent lipase, a 110 kDa pancreatic protein, in urines of healthy subjects.. Kidney Int.

[44] Cui S, Flyvbjerg A, Nielsen S, Kiess W, Christensen EI (1993). IGF-II/Man-6-P receptors in rat kidney: apical localization in proximal tubule cells.. Kidney Int.

[45] Haskell JF, Pillion DJ, Meezan E (1988). Specific, high affinity receptors for insulin-like growth factor II in the rat kidney glomerulus.. Endocrinology.

[46] Kornfeld S (1992). Structure and function of the mannose 6-phosphate/insulinlike growth factor II receptors.. Annu Rev Biochem.

[47] Oka Y, Rozek LM, Czech MP (1985). Direct demonstration of rapid insulin-like growth factor II Receptor internalization and recycling in rat adipocytes. Insulin stimulates 125I-insulin-like growth factor II degradation by modulating the IGF-II receptor recycling process.. J Biol Chem.

[48] Petersen CM, Nielsen MS, Nykjaer A, Jacobsen L, Tommerup N (1997). Molecular identification of a novel candidate sorting receptor purified from human brain by receptor-associated protein affinity chromatography.. J Biol Chem.

[49] Morris NJ, Ross SA, Lane WS, Moestrup SK, Petersen CM (1998). Sortilin is the major 110-kDa protein in GLUT4 vesicles from adipocytes.. J Biol Chem.

[50] Christensen EI, Birn H (2002). Megalin and cubilin: multifunctional endocytic receptors.. Nat Rev Mol Cell Biol.

[51] Verroust PJ, Birn H, Nielsen R, Kozyraki R, Christensen EI (2002). The tandem endocytic receptors megalin and cubilin are important proteins in renal pathology.. Kidney Int.

[52] Kerjaschki D, Farquhar MG (1983). Immunocytochemical localization of the Heymann nephritis antigen (GP330) in glomerular epithelial cells of normal Lewis rats.. J Exp Med.

[53] Yamazaki H, Saito A, Ooi H, Kobayashi N, Mundel P (2004). Differentiation-induced cultured podocytes express endocytically active megalin, a heymann nephritis antigen.. Nephron Exp Nephrol.

[54] Grone HJ, Walli AK, Grone E, Kramer A, Clemens MR (1990). Receptor mediated uptake of apo B and apo E rich lipoproteins by human glomerular epithelial cells.. Kidney Int.

[55] Eyre J, Ioannou K, Grubb BD, Saleem MA, Mathieson PW (2007). Statin-sensitive endocytosis of albumin by glomerular podocytes.. Am J Physiol Renal Physiol.

[56] Willnow TE, Goldstein JL, Orth K, Brown MS, Herz J (1992). Low density lipoprotein receptor-related protein and gp330 bind similar ligands, including plasminogen activator-inhibitor complexes and lactoferrin, an inhibitor of chylomicron remnant clearance.. J Biol Chem.

[57] Stefansson S, Chappell DA, Argraves KM, Strickland DK, Argraves WS (1995). Glycoprotein 330/low density lipoprotein receptor-related protein-2 mediates endocytosis of low density lipoproteins via interaction with apolipoprotein B100.. J Biol Chem.

[58] Kounnas MZ, Argraves WS, Strickland DK (1992). The 39-kDa receptor-associated protein interacts with two members of the low density lipoprotein receptor family, alpha 2-macroglobulin receptor and glycoprotein 330.. J Biol Chem.

[59] Tauris J, Ellgaard L, Jacobsen C, Nielsen MS, Madsen P (1998). The carboxy-terminal domain of the receptor-associated protein binds to the Vps10p domain of sortilin.. FEBS Lett.

[60] Kerjaschki D, Farquhar MG (1982). The pathogenic antigen of Heymann nephritis is a membrane glycoprotein of the renal proximal tubule brush border.. Proc Natl Acad Sci U S A.

[61] Stefano JE, Hou L, Honey D, Kyazike J, Park A (2009). In vitro and in vivo evaluation of a non-carbohydrate targeting platform for lysosomal proteins.. J Control Release.

[62] LeBowitz JH, Grubb JH, Maga JA, Schmiel DH, Vogler C (2004). Glycosylation-independent targeting enhances enzyme delivery to lysosomes and decreases storage in mucopolysaccharidosis type VII mice.. Proc Natl Acad Sci U S A.

[63] Prince WS, McCormick LM, Wendt DJ, Fitzpatrick PA, Schwartz KL (2004). Lipoprotein receptor binding, cellular uptake, and lysosomal delivery of fusions between the receptor-associated protein (RAP) and alpha-L-iduronidase or acid alpha-glucosidase.. J Biol Chem.

[64] Moestrup SK, Nielsen S, Andreasen P, Jorgensen KE, Nykjaer A (1993). Epithelial glycoprotein-330 mediates endocytosis of plasminogen activator-plasminogen activator inhibitor type-1 complexes.. J Biol Chem.

[65] Nykjaer A, Christensen EI, Vorum H, Hager H, Petersen CM (1998). Mannose 6-phosphate/insulin-like growth factor-II receptor targets the urokinase receptor to lysosomes via a novel binding interaction.. J Cell Biol.

[66] Nielsen MS, Madsen P, Christensen EI, Nykjaer A, Gliemann J (2001). The sortilin cytoplasmic tail conveys Golgi-endosome transport and binds the VHS domain of the GGA2 sorting protein.. EMBO J.

[67] Greenwood FC, Hunter WM, Glover JS (1963). The Preparation of I-131-Labelled Human Growth Hormone of High Specific Radioactivity.. Biochem J.

[68] Birn H, Fyfe JC, Jacobsen C, Mounier F, Verroust PJ (2000). Cubilin is an albumin binding protein important for renal tubular albumin reabsorption.. J Clin Invest.

